# Plasma Proteomics of COVID-19 Associated Cardiovascular Complications: Implications for Pathophysiology and Therapeutics

**DOI:** 10.21203/rs.3.rs-539712/v1

**Published:** 2021-06-08

**Authors:** Jason Roh, Robert Kitchen, J Sawalla Guseh, Jenna McNeill, Malika Aid, Amanda Martinot, Andy Yu, Colin Platt, James Rhee, Brittany Weber, Lena Trager, Margaret Hastings, Sarah Ducat, Peng Xia, Claire Castro, Bjarni Atlason, Timothy Churchill, Marcelo Di Carli, Patrick Ellinor, Dan Barouch, Jennifer Ho, Anthony Rosenzweig

**Affiliations:** Harvard Medical School; Harvard Medical School; Massachusetts General Hospital; Massachusetts General Hospital; Beth Israel Deaconess Medical Center BIDMC; Tufts University; Massachusetts General Hospital; Massachusetts General Hospital; Massachusetts General Hospital; Brigham and Women's Hospital; Massachusetts General Hospital; Massachusetts General Hospital; Tufts University; Massachusetts General Hospital; Massachusetts General Hospital; Massachusetts General Hospital; Massachusetts General Hospital; Brigham and Women's Hospital; The Broad Institute of MIT and Harvard; Beth Israel Deaconess Medical Center; Massachusetts General Hospital; Massachusetts General Hospital

**Keywords:** COVID-19, cardiovascular complications, disease severity, mortality

## Abstract

Cardiovascular complications are common in COVID-19 and strongly associated with disease severity and mortality. However, the mechanisms driving cardiac injury and failure in COVID-19 are largely unknown. We performed plasma proteomics on 80 COVID-19 patients and controls, grouped according to disease severity and cardiac involvement. Findings were validated in 305 independent COVID-19 patients and investigated in an animal model. Here we show that senescence-associated secretory proteins, markers of biological aging, strongly associate with disease severity and cardiac involvement even in age-matched cohorts. FSTL3, an indicator of Activin/TGFβ signaling, was the most significantly upregulated protein associated with the heart failure biomarker, NTproBNP (β = 0.4;p_adj_=4.6x10^−7^), while ADAMTS13, a vWF-cleaving protease whose loss-of-function causes microvascular thrombosis, was the most downregulated protein associated with myocardial injury (β=−0.4;p_adj_=8x10^−7^). Mendelian randomization supported a causal role for ADAMTS13 in myocardial injury. These data provide important new insights into the pathophysiology of COVID-19 cardiovascular complications with therapeutic implications.

## Introduction

Since its discovery in December 2019, the severe acute respiratory syndrome coronavirus 2 (SARS-CoV-2) has resulted in ~ 160 million cases of coronavirus disease 2019 (COVID-19) and nearly 3.3 million deaths globally.^1^

Although viral pneumonia is the predominant clinical manifestation of severe COVID-19, cardiovascular complications occur frequently and are independently associated with morbidity and mortality. Among hospitalized COVID-19 patients, myocardial injury occurs in ~ 20–62%,^2-6^ vascular thrombosis in ~ 16%,^7^ and acute heart failure in ~ 2.5%.^8^ Acute heart failure in COVID-19 often occurs in those without preexisting heart failure, and is associated with mortality rates approaching 47%.^8^ Myocardial injury, the most common cardiac complication in COVID-19, also remains a strong predictor for adverse outcomes and is associated with ≥ 2-fold increase of in-hospital mortality.^6^

There are multiple potential contributors to cardiac injury and dysfunction in COVID-19.^9-13^ While myocardial injury, indicated by elevations in circulating cardiac troponins, is common in COVID-19,^2-5,14,15^ both Type I myocardial infarction caused by epicardial coronary artery occlusion and myocarditis appear rare,^11,12^ suggesting other mechanisms drive most COVID-19-related cardiac injury and failure. We systematically examined circulating proteins in controls and COVID-19 patients to identify potential drivers of cardiovascular complications. These studies support inferences into the pathogenesis of COVID-19-induced cardiac injury and implicate pathways amenable to intervention in this process.

## Results

### Characteristics of discovery study participants

The cohort for the case control plasma proteomics discovery study had a mean age of 64 years and 41% women (Table 1). There were no significant differences in age or sex between COVID-19 patients and controls, though controls included more white subjects and there was a non-significant trend of higher age in moderate COVID-19 patients with cardiac involvement due to limited samples available for matching. Controls had higher body mass index, but less coronary artery disease, diabetes, and cancer. There were no differences in baseline hypertension, hyperlipidemia, myocardial infarction, stroke, liver or pulmonary disease, except for a higher prevalence of obstructive sleep apnea in controls. Among moderate COVID-19 patients, those with cardiac involvement had more chronic kidney disease and baseline anticoagulation use. In severe COVID-19 patients, cancer was more prevalent in those with higher levels of cardiac involvement. Cardiac involvement in COVID-19 patients was generally associated with higher high-sensitivity cardiac troponin T (hsTnT), N-terminal pro-B natriuretic peptide (NTproBNP), C-reactive protein (CRP), and D-dimer levels (Supplementary Table 1). There was no significant difference in PE and DVT, although event rates were relatively low. Severe COVID-19 patients with high degrees of cardiac involvement had the highest in-hospital mortality (33%) (Supplementary Table 1).

### Plasma proteins associated with senescence and microvascular inflammation increase with worsening COVID-19 severity and cardiac involvement

**Figure 1:**
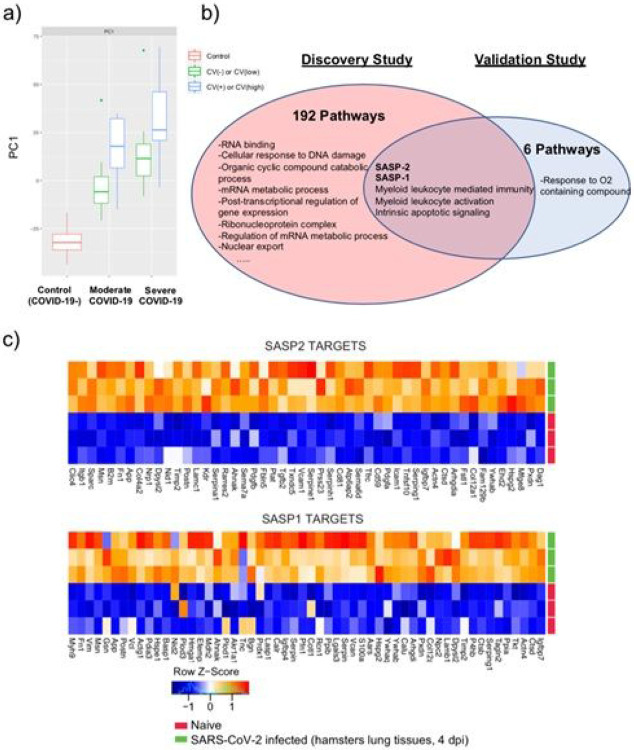
Senescence associated secretory phenotype is associated with COVID-19 severity and cardiac involvement. a) Principal component analysis of the discovery proteomics study with boxplots displaying unsupervised clustering of the 80 patient plasma samples based on PC1 proteins. b) Venn diagram displaying overlapping pathways associated with increasing COVID-19 severity in the discovery and validation cohorts. In the discovery cohort, enrichment analysis was done with PC1 proteins (Supplementary Table 2). In the validation cohort, pathway analysis was performed using Day 0 proteomic profiles regressed on maximum WHO disease severity per patient (Supplementary Table 8). c) Heat maps displaying differential expression of the 50 most significantly regulated genes in the SASP-1 or SASP-2 gene sets in lungs from hamsters infected with SARS-CoV2 versus naïve controls. Tissue samples were collected four days after infection. n=3/group. SASP = senescence associated secretory phenotype.

To first determine if the plasma proteomic profiles distinguish subjects according to COVID-19 disease severity and/or cardiac involvement, we performed unsupervised principal component analysis in the discovery cohort. Strikingly, the first principal component (PC1), representing the maximum variance in the dataset, clustered most subjects into their pre-specified group (Fig. 1a). This suggests the plasma proteome not only distinguishes between subjects with and without COVID-19, but can also accurately discriminate among varying COVID-19 disease severity and cardiac involvement. Notably, cardiac TnT and NTproBNP, proteins used to assign subjects to the different groups, were not the dominant proteins driving PC1 (Supplementary Fig. 2). Rather, enrichment analysis of PC1 proteins identified innate immune responses, senescence, RNA processing, and cell injury as the most significant biological processes that discriminate among these five groups (Fig. 1b; Supplementary Table 2).

Interestingly, despite groups being age-matched, proteins in a senescence-associated secretory phenotype (SASP-1) set (Supplementary Table 3)^16^, markers of biological aging, emerged as the most upregulated process (normalized enrichment score [NES] = 2.2; p_adj_=0.01) (Supplementary Table 2). To confirm this finding, an additional SASP-2 set was generated from a literature search of all published SASP proteins (Supplementary Table 3) and was also highly enriched in PC1 (NES = 2.0; p_adj_=0.01). Subgroup analyses consistently showed SASP-2 to be the most significantly upregulated process associated with cardiac involvement in both moderate (NES = 1.6, p_adj_=0.03) and severe (NES = 1.7, p_adj_=0.02) COVID-19 (Supplementary Tables 4-7). In an independent validation cohort of 305 COVID-19 patients, the two SASP protein sets were the pathways most significantly associated with disease severity (Fig. 1b; Supplementary Table 8). Interestingly, in transcriptional profiles of lungs from hamsters infected with SARS-CoV2, we also saw marked enrichment of both SASP-1 (NES = 1.8; FDR q-value = 0.001) and SASP-2 (NES = 2.2; FDR q-value < 0.001) compared to age-matched non-infected controls (Fig. 1c, Supplementary Table 9), suggesting that this age-related cellular senescence profile is induced by COVID-19.

**Figure 2:**
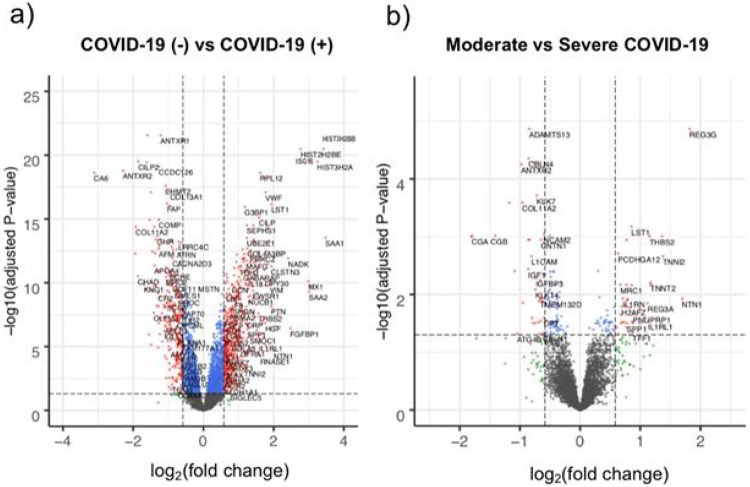
Differentially expressed plasma proteins associated with COVID-19 infection and disease severity. a) Volcano plot displaying differentially expressed plasma proteins in the discovery study when comparative analysis was done between COVID-19 patients (n=54) versus controls (n=26). b) Volcano plot displaying differentially expressed plasma proteins in the discovery study when comparing COVID-19 patients with moderate (n=25) versus severe (n=29) disease. Proteins with significant (padj<0.05) differences in abundance (non-significant proteins colored black) with an absolute change greater than 1.5-fold are colored red (proteins with more modest fold-change are colored blue). For all analyses, p-values were adjusted for multiple hypothesis testing using the Benjamini & Hochberg method.

To identify circulating proteins associated with COVID-19 infection, we next compared plasma proteomes of COVID-19 patients (n = 54) to non-infected controls (n = 26). Consistent with the microvascular pathology seen in COVID-19,^9-13,17^ plasma proteomic signals strongly suggested endothelial injury. The most upregulated plasma proteins in COVID-19 were extracellular histones (Fig. 2a, Supplementary Table 10), markers of neutrophil extracellular traps implicated in COVID-19-related microvascular inflammation and thrombosis.^17^ Furthermore, von Willebrand Factor (vWF), a clotting factor secreted by activated endothelial cells, was one of the most significantly elevated circulating proteins in COVID-19 (341% higher; p_adj_=8.3x10^−18^, Fig. 2a, Supplementary Table 10).

To identify proteins correlated with COVID-19 severity, we compared patients with moderate (n = 25) versus severe (n = 29) COVID-19 and found 171 plasma proteins differentially expressed between the groups (Fig. 2b, Supplementary Table 11). The most significantly increased protein in severe COVID-19 was regenerating islet-derived protein 3 gamma (REG3G; 353% higher; p_adj_=1.4x10^−5^), an antimicrobial protein secreted by pulmonary and gut epithelium.^18,19^ Interestingly, two of the 10 most significantly downregulated proteins were neural cell adhesion molecules (NCAM1 and NCAM2), which were 38% and 34% lower in patients with severe COVID-19, respectively (p_adj_=6.6x10^−5^ and 9.0x10^−4^). NCAMs regulate axonal outgrowth and olfactory development,^20^ raising the possibility of a role in the anosmia phenotype sometimes seen in COVID-19. The most striking finding, however, was a 44% reduction in ADAMTS13 (A Disintegrin And Metalloproteinase with a Thrombospondin type I motif, member 13), which was the most significantly downregulated of the 4996 protein analytes in severe COVID-19 (p_adj_=1.4x10^−5^). ADAMTS13 is a secreted protease that inhibits thrombosis by cleaving vWF multimers, and its deficiency is a known cause of spontaneous microvascular clot formation and thrombotic thrombocytopenia purpura (TTP).^21^ vWF/ADAMTS13 ratios progressively increased with worsening COVID-19 severity (Supplementary Fig. 3), suggesting that a deficiency in ADAMTS13 levels relative to its known substrate occurs with increased COVID-19 severity. Furthermore, ADAMTS13 levels inversely correlated with clinical D-dimer levels in COVID-19 patients (r=−0.7, p < 0.001, Supplementary Fig. 4), suggesting that the overall thrombotic burden in COVID-19 patients is higher in those patients with lower ADAMTS13 levels.

### ADAMTS13 deficiency in COVID-19 related myocardial injury

**Figure 3:**
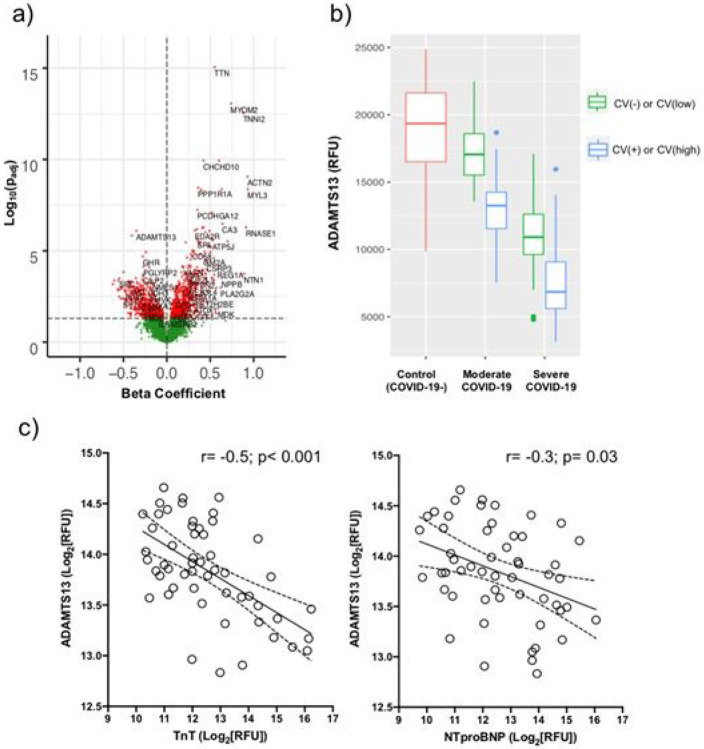
Inverse association of ADAMTS13 with myocardial injury in COVID-19. a) Volcano plot displaying differentially expressed plasma proteins in the discovery study after regression on cardiac TnT levels. Beta coefficient is plotted on the X-axis showing magnitude and direction of correlation with TnT. Red = significant (padj<0.05). Green = non-significant. P-values were adjusted for multiple hypothesis testing using the Benjamini & Hochberg method. b) Boxplots displaying median and inter-quartile range of plasma ADAMTS13 levels according to disease severity and cardiac involvement in the discovery cohort. c) Pearson correlations of ADAMTS13 levels with cardiac biomarkers of myocardial injury (cardiac TnT) and myocardial stress (NTproBNP) in COVID-19 patients in the discovery cohort. Solid line represents best fit line after simple linear regression with dashed lines representing the 95% confidence interval.

**Figure 4:**
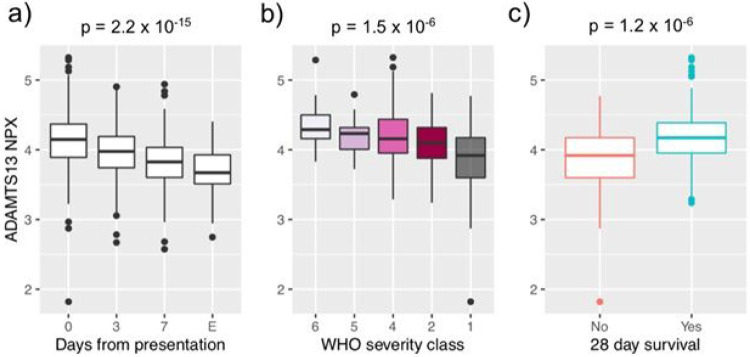
Association of lower ADAMTS13 levels with disease severity and mortality in COVID-19. Boxplots of ADAMTS13 plasma levels in the MGH Emergency Department validation cohort (n=305 COVID-19 patients) according to a) days from initial presentation or at event, E, marking significant clinical deterioration, b) maximum COVID-19 severity (WHO classification), or c) 28-day survival. Boxplots represent median with inter-quartile range. NPX = normalized protein expression.

To gain insight into the drivers of myocardial injury in COVID-19, we regressed our datasets on cardiac troponin (TnT) SOMAmer levels, which strongly correlated with clinical hsTnT concentrations (r = 0.8, p = 3.1x10^−13^, Supplementary Fig. 5). 1,143 proteins differed significantly after regression on TnT (Fig. 3a, Supplementary Table 12). The proteins with the most significant positive association with cardiac TnT were mostly intracellular myocyte proteins, likely reflecting myocardial necrosis. Interestingly, the protein with the most significant negative association with cardiac TnT was ADAMTS13 (β coefficient= −0.4; p_adj_=8x10^−7^). Given the marked and progressive decline in ADAMTS13 levels observed with increasing cardiac involvement along with its strong correlation with TnT (Fig. 3b and 3c), we looked to see if a similar phenomenon was occurring in the COVID-19 patients from the MGH Emergency Department validation cohort. Indeed, in these 305 patients, plasma ADAMTS13 levels displayed a similarly robust inverse correlation with cardiac troponin I (r=−0.4, p = 2x10^−14^, Supplementary Fig. 6) and decreased further as COVID-19 progressed (p = 2.2x10^−15^; Fig. 4a). Moreover, lower ADAMTS13 levels on presentation were associated with overall disease severity (p = 1.5x10^−6^) and 28-day mortality (p = 1.2x10^−6^) (Fig. 4b and 4c).

**Figure 5:**
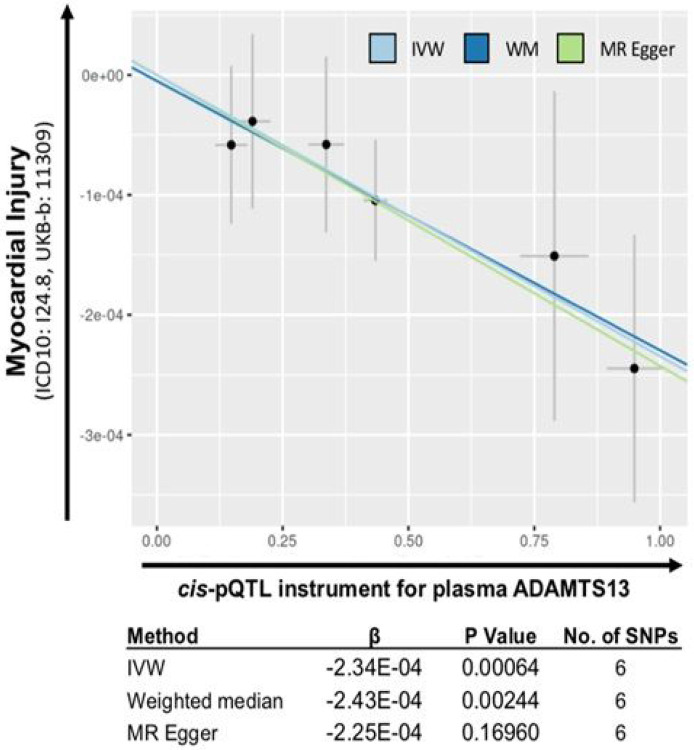
Mendelian randomization analysis of cis-pQTLs in ADAMTS13 gene infers causality in myocardial injury. Scatterplot of SNP effects on ADAMTS13 plasma levels (pQTLs) and clinically diagnosed myocardial injury in the UK Biobank general population sample (n=463,010; 269 cases and 462,741 controls). The slope of each line corresponds to the estimated Mendelian randomization effect per method. (Light blue= Inverse variance-weighted method; Dark blue= Weighted median method; Green= MR Egger method). This demonstrates that genetically determined decreases in ADAMTS13 levels are quantitatively associated with increasing risk of myocardial injury, and support a causal role of ADAMTS13 in this context.

To assess a potential causal role of decreased ADAMTS13 in myocardial injury, we performed Mendelian randomization (MR) analysis in 463,010 subjects in the UK BioBank using intronic cis-pQTLs, genetic determinants of ADAMTS13 protein levels that map to the gene itself. Since these genetic alleles are inherited independent of confounders, a positive association provides evidence that ADAMTS13 levels are in a causal pathway with cardiac injury, even outside the severe effects seen in TTP. Indeed, we found that lower genetically-determined levels of plasma ADAMTS13 were associated with higher likelihood of clinically diagnosed myocardial injury in a large, general population (IVW β= −2.34x10^−4^; p = 6.4x10^−4^) (Fig. 5 and Supplementary Fig. 7), suggesting that reduced ADAMTS13 levels increase vulnerability to cardiac injury.

**Figure 6:**
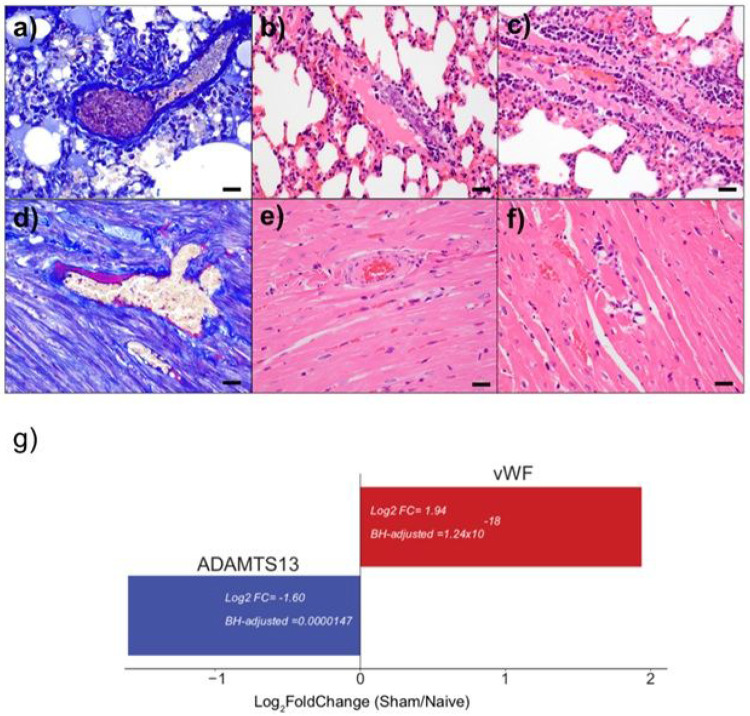
Pulmonary and cardiac vascular thrombus formation and decreased ADAMTS13 expression in SARS-CoV2 infected Syrian hamsters. Hamsters were infected with 5x105 TCID50 SARS CoV-2 and euthanized on day 4 following challenge. a) thrombus, pulmonary vein. b) endothelialitis, pulmonary vein. c) endothelialitis, pulmonary artery. d) fibrin deposition along endothelium, cardiac vein. e) fibrin deposition, cardiac artery with adjacent myodegeneration. f) focal myocarditis and myodegeneration. (a,c) Carstairs stain. (b, c, e, f) hematoxylin and eosin. Scale bars= 20 microns. g) Relative change in mRNA levels of ADAMTS13 and its substrate, vWF, in lung tissue from hamsters infected with SARS-CoV2 (n=3) compared to naïve uninfected controls (n=3). Tissues samples were collected at four days post-infection and mRNA levels quantified used bulk RNAseq. P-values were adjusted for multiple hypothesis testing using the Benjamini & Hochberg method.

To further investigate why circulating ADAMTS13 levels are lower in severe COVID-19 patients, we looked to see if ADAMTS13 gene expression changed in the SARS-CoV2 infected hamsters. Similar to humans, hamsters have severe endothelialitis associated with SARS-CoV2 infection^9,22^. We found evidence of vascular thrombus formation in the lungs and small vessel fibrin deposition and cardiomyocyte degeneration consistent with microvascular compromise in infected animals (Fig. 6a-f). Compared to naïve controls, ADAMTS13 expression was 67% lower in SARS-CoV2 infected animals (p_adj_=1x10^−5^; Fig. 6c), suggesting that at least part of the decrease in circulating ADAMTS13 seen in those with severe COVID-19 is through decreased synthesis of this anti-thrombotic protein. This was associated with a 3.8-fold increase in vWF expression (p_adj_=1.2x10^−18^; Fig. 6c).

### Increased TGFβ superfamily signaling associated with the heart failure biomarker, NTproBNP, in COVID-19

**Figure 7:**
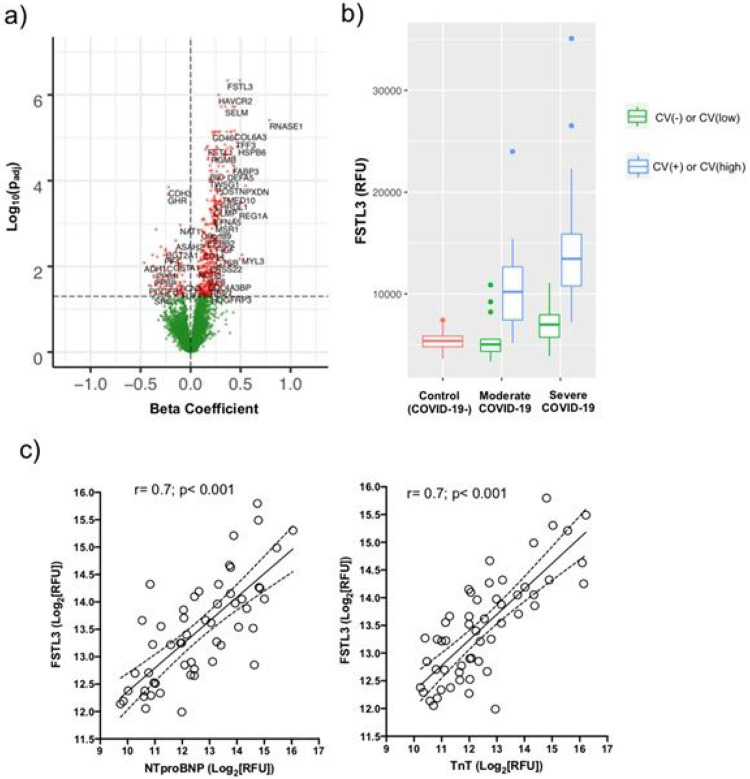
Association of TGFβ superfamily signaling with heart failure biomarker in COVID-19. a) Volcano plot displaying differentially expressed plasma proteins in the discovery study after regression on NTproBNP levels. Beta coefficient is plotted on the X-axis showing magnitude and direction of correlation with NTproBNP. Red = significant (padj<0.05). Green = non-significant. P-values were adjusted for multiple hypothesis testing using the Benjamini & Hochberg method. b) Boxplots displaying median and inter-quartile range of plasma FSTL3 levels according to disease severity and cardiac involvement in the discovery cohort. c) Pearson correlations of FSTL3 levels with cardiac biomarkers for myocardial stress (NTproBNP) and myocardial injury (cardiac TnT) in the discovery cohort. Solid line represents best fit line after simple linear regression with dashed lines representing the 95% confidence interval.

Although cardiac microvascular thrombosis is likely a major contributor to myocardial injury in COVID-19, it is only identified in a subset of patients.^11,12^ Non-ischemic etiologies, such as right ventricular strain, myocarditis, and stress cardiomyopathy, are other sources of cardiac injury and dysfunction in COVID-19, which often result in high circulating levels of both cardiac troponins and the heart failure biomarker, NTproBNP.^11,23,24^ While ADAMTS13 showed an inverse relationship with NTproBNP, the correlation was modest (r=−0.3; p = 0.03, Fig. 3c), suggesting other processes are likely more substantial contributors to myocardial stress in COVID-19. After regression on NTproBNP Somamer levels, which strongly correlated with the clinical assay (r = 0.8; p = 3.8x10^−12^; Supplementary Fig. 8), 526 proteins were significantly different. Of these, follistatin-like 3 (FSTL3), a marker of TGFβ and Activin receptor signaling,^25^ displayed the most significant positive association with NTproBNP (β coefficient = 0.4; p_adj_=4.6x10^−7^; Fig. 7a, Supplementary Table 13). FSTL3 levels correlated strongly with both NTproBNP and cardiac TnT (r = 0.7, p < 0.001 for both), and were markedly higher in both moderate and severe COVID-19 patients with cardiac involvement compared to their respective controls (Figs. 7b and 7c, Supplementary Tables 4 and 6).

**Figure 8:**
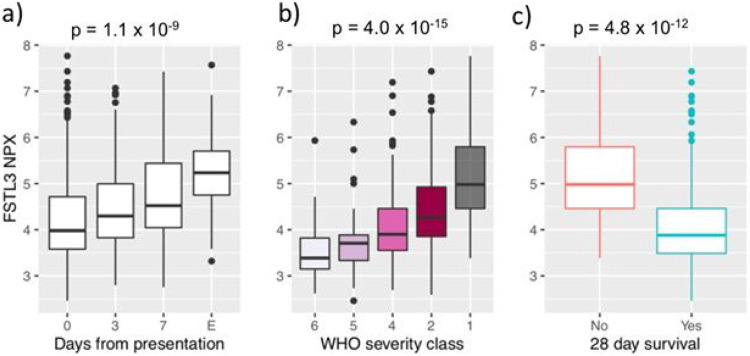
Association of TGFβ superfamily signaling with disease severity and mortality in COVID-19. Boxplots of FSTL3 levels in the MGH Emergency Department validation cohort according to a) time from clinical presentation in days or at event, E, marked by significant clinical deterioration, b) maximum COVID-19 severity by WHO classification, or c) 28-day survival. Boxplots represent median with inter-quartile range. NPX = normalized protein expression.

The association between FSTL3 and NTproBNP levels robustly validated in the independent MGH Emergency Department cohort. FSTL3 levels showed similarly powerful correlations with NTproBNP (r = 0.7; p < 2x10^−16^) and cardiac TnT (r = 0.6; p < 2x10^−16^; Supplementary Fig. 9) and increased with disease duration (p = 1.1x10^−9^; Fig. 8a). Moreover, presenting FSTL3 levels strongly associated with both disease severity (p = 4.0x10^−15^) and 28-day mortality (p = 4.8x10^−12^) in COVID-19 (Figs. 8b and 8c).

MR analysis was unable to be performed for FSTL3 because only a single pQTL (rs12986335) has been identified for this protein and this pQTL did not reach genome-wide significance. Because FSTL3 is an indirect marker of increased TGFβ and Activin receptor signaling rather than part of the causal pathway, we would not anticipate a causal relationship between FSTL3 and heart failure. Since expression of FSTL3 is induced by activation of TGFβ and Activin receptors, which can be initiated by multiple ligands of the TGFβ superfamily, including TGFβ, Activins, bone morphogenic proteins (BMPs), and growth differentiation factors (GDFs),^25,26^ we looked to see which of these ligands might be contributing to the increased circulating FSTL3 observed in COVID-19. Fourteen of the relevant ligands are measured on the SOMAscan platform, and twelve of these positively correlated with FSTL3 (Supplementary Fig. 10). To determine which of these are likely responsible for the association of FSTL3 with increased myocardial stress in COVID-19, we exposed isolated cardiomyocytes to the various ligands, of which only Activins and TGFβ robustly increased cardiomyocyte FSTL3 expression (Supplementary Fig. 11). Of these proteins, TGFβ1 was the most significantly increased in COVID-19 patients (74% higher than controls, p_adj_=7.2x10^−11^), suggesting it may be the primary circulating ligand responsible for the increase in FSTL3 observed in COVID-19 patients with elevated NTproBNP. Although we were unable to determine if TGFβ1 expression is directly increased in the hearts of those with COVID-19, RNAseq profiles of lungs from hamsters infected with SARS-CoV2 indicated that TGFβ1 signaling is indeed upregulated in animals with severe infection (NES = 1.5; FDR q-value = 0.03, Supplementary Fig. 12).

## Discussion

Cardiovascular complications are common in COVID-19 and are strongly associated with disease severity and mortality. Despite this, the principal drivers of cardiac injury and dysfunction remain unclear and no effective therapies for these complications of COVID-19 have been identified. To gain mechanistic insights into the underlying pathophysiology, we systematically compared circulating plasma proteins in controls and COVID-19 patients across a range of disease severity and cardiac involvement. Key findings from our 80 subject discovery cohort were not only validated in a distinct, longitudinal cohort of 305 COVID-19 patients, but also investigated in an experimental animal model of severe COVID-19. Our analyses identified alterations in two biological pathways that strongly associated with cardiac injury in COVID-19: a reduction in the anti-thrombotic protein, ADAMTS13, and an increase in TGFβ superfamily signaling, previously linked to cardiac dysfunction, injury, and fibrosis.^25,27^ These changes, along with an increase in senescence-associated secretory proteins, were also associated with disease severity and mortality. Notably, these pathways are potentially amenable to therapeutic intervention with agents already in clinical trials or FDA approved for other indications.^28,29^

Particularly striking was the marked ADAMTS13 reduction that progressed with COVID-19 duration and severity. Of the 4996 protein analytes assessed in our discovery study, ADAMTS13 was not only the most significantly decreased protein in severe COVID-19, but also displayed the strongest inverse association with myocardial injury. Autopsies have found evidence of microvascular thrombosis in multiple organs, including the heart, in COVID-19 patients.^9-12,30^ Given its role in thrombotic microangiopathies,^21^ the possibility that an acquired ADAMTS13 deficiency or local imbalance with its substrate, vWF, could be driving thrombotic complications in COVID-19 has been raised.^30-32^ Our data provide new evidence supporting an important role for reduced ADAMTS13 in COVID-19 related myocardial injury. Mendelian randomization analysis of genetic variants within the ADAMTS13 gene strongly support a causal relationship between plasma ADAMTS13 protein levels and myocardial injury. Furthermore, the decreased mRNA expression of ADAMTS13 seen in the hamster model after SARS-CoV2 infection suggests that the decline in circulating ADAMTS13 in severe COVID-19 is at least partially due to a decrease in its synthesis. Given recent safety concerns of therapeutic systemic anticoagulation in critically ill COVID-19 patients^33^, specifically replenishing ADAMTS13 in patients with low levels could provide a safer, targeted, and potentially more effective strategy for preventing vascular thrombosis in patients with severe COVID-19. Notably, recombinant ADAMTS13 has demonstrated safety in patients with congenital TTP,^28^ and recent pilot testing of ex vivo plasma samples from patients with severe COVID-19 suggests it can effectively reduce the heightened vWF activity and increased ultra-high molecular weight vWF multimers observed in COVID-19^34^. Based on our collective findings in humans and animals with COVID-19, we propose that targeted ADAMTS13 repletion warrants evaluation as a tailored approach to preventing myocardial injury and possibly other thromboembolic complications in patients with severe COVID-19 and reduced ADAMTS13 levels.

In addition to microvascular thrombosis, other non-ischemic etiologies, including stress cardiomyopathy and right ventricular strain, also contribute to myocardial necrosis in COVID-19, often resulting in substantial increases in not only cardiac troponins but also the heart failure biomarker, NTproBNP.^11,23,24^ Indeed in our analysis of the plasma proteome, we found the dominant proteomic signal associated with NTproBNP was not ADAMTS13, but rather FSTL3, a marker of TGFβ superfamily signaling.^25^ FSTL3 has been identified as one of only four circulating proteins (out of 1310 assayed) that potentially distinguishes between patients with acute stress cardiomyopathy in comparison to acute myocardial infarction.^35^ It is possible that cardiac dysfunction driven by TGFβ superfamily signaling is acutely reversible as seen in stress cardiomyopathy, consistent with some clinical observations in COVID-19.^36^ However, the close association of TGFβ superfamily signaling with fibrosis raises the possibility that these pathways may also contribute to some of the long-term sequelae beginning to be appreciated in survivors of severe COVID-19. Importantly, multiple TGFβ superfamily inhibitors, including those currently being investigated in clinical trials, have shown benefits in preclinical models of heart failure, as well as acute lung injury.^25,37-39^ While these data suggest that this class of reagents could attenuate cardiac and pulmonary injury in COVID-19, given the immunomodulatory roles of TGFβ,^40^ careful patient selection and optimal timing of such interventions will be critical to maximizing therapeutic benefit while minimizing adverse effects on immune responses to the virus.

Lastly, our proteomics analysis detected a novel association between senescence associated secretory proteins, markers of biological aging, with disease severity and cardiac involvement in COVID-19. While age is a recognized risk factor for adverse COVID-19 outcomes, since study subjects in our discovery study were mostly age-matched, this finding suggests that *biological* aging may represent a more accurate marker of COVID-19 severity than *chronological* aging alone. Indeed, in the validation cohort, a subject’s SASP profile on presentation was the most significant pathway associated with disease severity over the subsequent 28 days. Furthermore, the marked SASP enrichment seen in hamsters with severe COVID-19, suggests that SARS-CoV2 infection induces an increase in SASP production. This is consistent with the increase in circulating SASP proteins observed in patients with more severe COVID-19, which interestingly has also been linked to an increase in vascular thrombosis.^41^ Of course, these possibilities are not mutually exclusive. It is possible that pre-existing biological aging, reflected in circulating SASP proteins, puts patients at higher risk for severe COVID-19 and that SARS-CoV2 infection also induces cellular senescence, further increasing circulating SASP proteins in a positive feedback loop. While targeting senescence has been proposed as a potential therapeutic strategy for severe COVID-19^42,43^, these are the first data to report an increase in SASP profiles in COVID-19 patients that tracks with disease severity. Understanding the role of senescence in COVID-19 pathophysiology could be important in the context of therapeutic development and as vaccine development and rollout continue over the coming year.^44^

Several limitations of our study are worth noting. First, observational data cannot definitively establish causality. In the case of ADAMTS13, however, additional inferences from genetics strongly support a pathogenic role. Further studies of SARS-CoV2 infection in Syrian hamsters also mechanistically link viral infection to reduced ADAMTS13 expression. Second, the discovery study sample sizes were relatively small. In this context, the robust validation of our key findings in a larger dataset generated using an independent proteomic technology is reassuring. Finally, while promising candidates were identified that might be targeted to mitigate COVID-19 cardiac complications, these hypotheses require rigorous additional testing. Importantly, our clinical observations strongly resonate with findings in the hamster model of severe COVID-19, further supporting an important role of these processes in COVID-19 pathophysiology. These findings also suggest this model could provide a preclinical tool for testing the safety and therapeutic efficacy of currently available reagents targeting these candidates as a prelude to clinical trials.

In summary, systematic analysis of circulating plasma proteins revealed important insights in COVID-19 cardiovascular pathophysiology and provides a foundation for novel therapeutic approaches to prevent or treat cardiac complications of COVID-19. Further investigation is warranted to determine if restoring ADAMTS13 levels, inhibiting TGFβ superfamily signaling, or modulating senescence can mitigate cardiovascular complications and improve outcomes in COVID-19 patients.

## Methods

### Discovery Study:

To identify proteins that change with cardiac involvement in COVID-19, a case-control plasma proteomics study was performed in 80 subjects whose plasma samples were available from the multi-institutional Massachusetts Consortium on Pathogen Readiness and the Mass General Brigham (MGB) Biobank. COVID-19 subjects were recruited and plasma samples were collected with informed consent and IRB approval between March and June 2020 at Massachusetts General Hospital and Brigham and Women’s Hospital in Boston, MA. Subjects were chosen based on data from their electronic medical records, to fill five pre-specified groups representing a range of disease severity and cardiac involvement. To reduce the confounding influence of factors previously linked to COVID-19 severity, groups were matched as much as possible for age, sex, and race. Patients enrolled in immunomodulator clinical trials or receiving such agents off-label were excluded. Study groups included non-COVID-19 controls; COVID-19 patients with moderate disease (hospitalized, but not requiring intensive care unit [ICU] level of care) with or without cardiac involvement; and COVID-19 patients with severe disease (requiring ICU care) with low or high degrees of cardiac involvement (Table 1). In patients with moderate COVID-19, cardiac involvement was defined as at least one of the following near time of plasma collection: a high-sensitivity cardiac troponin T (hsTnT) > 14ng/L, N-terminal pro-B natriuretic peptide (NTproBNP) > 1,800pg/ml, and/or cardiac dysfunction documented by transthoracic echocardiography (left ventricular ejection fraction [LVEF] < 50%, regional wall motion abnormality, right ventricular systolic dysfunction, or > 25% relative decrease from prior LVEF). The hsTnT threshold of 14 ng/L is consistent with the Fourth Universal Definition of Myocardial Infarction,^45^ which defines myocardial injury as a troponin concentration greater than the 99th percentile upper reference limit. For severe COVID-19 patients in the ICU, who have a high prevalence of mild troponin elevation, we used a hsTnT threshold of 20 ng/L to distinguish between low and higher levels of myocardial injury, while the remainder of the criteria were the same. Clinical outcomes included intubation, vasopressor requirement, neuromuscular blockade use, sedation, renal replacement therapy (RRT), pulmonary embolism (PE), deep venous thrombosis (DVT), and in-hospital mortality.

#### Plasma Proteomic Profiling

All blood samples obtained from subjects in the discovery study were anticoagulated with EDTA and collected plasma was frozen at −80C and underwent two freeze-thaw cycles before proteomic analysis. An aptamer-based proteomics platform (version 4; Somalogic) was used to measure relative levels of 4996 analytes, corresponding to 4730 distinct human proteins.

### Clinical Validation Study:

Key findings in the discovery proteomics study were validated in a publicly available, independent plasma proteomics dataset from the MGH Emergency Department COVID-19 Cohort (Filbin, Goldberg, Hacohen) analyzed using Olink, an antibody-based proteomics platform^46^. This cohort included 305 COVID-19 patients. Patients who were hospitalized had serial plasma samples collected during their hospitalization on days 0, 3, and 7. For 36 patients, an additional sample was collected on day E, corresponding to a significant clinical deterioration event. Maximum disease severity for each subject within 28 days of presentation was determined according to the World Health Organization (WHO) Ordinal Outcomes Scale (1 = death within 28 days, 2 = Intubated, ventilated, survived to 28 days, 3 = Non-invasive ventilation or high-flow nasal cannula, 4 = Hospitalized, supplementary O2 required; 5 = Hospitalized, no supplementary O2 required; 6 = Not hospitalized).

### Mendelian Randomization of ADAMTS13 cis-pQTLs:

Automated 2-sample Mendelian randomization (MR) was performed through an open-source application, MR-Base (www.mrbase.org), that systematically supports causal inference interrogation with summary-level data^47^. A multivariant instrument was constructed to genetically estimate protein levels of ADAMTS13 using locus-wide significant pQTLs acquired from PhenoScanner (www.phenoscanner.com). All locus wide intronic significant pQTLs (p < 3.88x10^−4^) were ranked by effect size and pruned for independence (R^2^ < 0.1). Rare variants were excluded (MAF > 0.01). For each direction of influence, we combined the MR effect estimates from each instrument SNP using inverse variance weighted (IVW) meta-analysis to obtain a meta-analytic estimate. Sensitivity analyses were performed through the standard and additional evaluation of a weighted median and an MR-Egger estimate. Given an experimentally derived hypothesis that COVID-19 results in cardiac injury without infarction, we sought the most proximate ICD10 code for acute myocardial injury (ICD: I24.8, Other forms of acute ischemic heart disease). Summary statistics were acquired from UKB-b:11309 (n = 463,010 with the dataset containing 269 cases and 462,741 controls). Outcome summary statistics were accessed 9/20/2020 in MR-Base. See Supplementary Fig. 1 for a detailed flow diagram of the MR procedure used.

#### Golden Syrian Hamster COVID-19 Model

Cardiac and pulmonary tissue from six golden Syrian hamsters (Envigo), ages 10–12 week old, from our prior work^22^ were used to further investigate candidate targets from the human discovery study. All animals were housed at Bioqual and studies were approved by their Institutional Animal Care and Use Committee. Animals were divided into two groups, naïve (uninfected) controls and SARS-CoV2 infected (n = 3/group). SARS-CoV2 infection was performed using previously published methods^22^. Briefly, animals in the infected group were each challenged with 5.0x10^5^ TCID_50_ (6x10^8^ vp, 5.5 × 10^4^ plaqueforming units (PFU)) SARS-CoV2, derived from USA-WA1/2020 (NR-52281, BEI Resources). Each animal in the infected group received 100 ml virus via intranasal administration. On day 4 post-infection, all animals were euthanized. Heart and lung samples were either fixed in 4% paraformaldehyde for histologic staining or homogenized in QIAzol (Qiagen) and stored at −80C for RNA sequencing. Histological tissue processing, Carstair’s method, and hematoxylin and eosin staining protocols were performed using previously published methods^22,48^. RNA was extracted from frozen lung tissue using the miRNeasy kit (Qiagen) with on-column DNase digestion, and quality was assessed using an Agilent Bioanalyzer. Library preparation was done using the SMARTer Stranded Total RNA-Seq V2 Pico Input Mammalian kit (Taara Bio). Libraries were validated with an Agilent 4200 TapeStation, and sequenced on an Illumina NovaSeq6000 at 100SR, targeting 25–30 million reads per sample.

#### FSTL3 Induction in Isolated Cardiomyocytes

Neonatal rat cardiomyocyte isolation, culture, and gene expression studies were performed using previously published methods^25^. Briefly, isolated cardiomyocytes were cultured in serum-free medium and incubated for 18 hours with 10ng/ml of various recombinant ligand proteins (Peprotech or R&D Systems) known to bind TGFβ receptors or Activin type II receptors (ActRII). RNA was extracted using Trizol, and polymerase chain reaction was done using SYBR green and standard amplification protocols. Relative changes in gene expression were measured using the ΔΔCT method.

#### Bioinformatic and Statistical Analyses

For analysis of the SOMAscan proteomics discovery study, median normalized relative abundances of the 4996 analytes were imported into *R*(v3.6.1) using the *SomaDataIO* package. Principal component analysis (PCA) was performed on log-transformed values using R’s *prcomp* function, differential protein abundance using *limma* (version 3.40.6), and protein enrichment using *clusterProfiler* (version 3.12.0). Pathway analysis was done using Gene Ontology data (MSigDB), appended with two curated senescence associated secretory phenotype (SASP) pathways. For gene-set enrichment analyses (GSEA), proteins were ranked by t-statistic and 50,000 permutations used to compute the nominal enrichment p-values. For all analyses, p-values were adjusted for multiple comparisons using the Benjamini & Hochberg method.

For validation using the data released by Filbin and colleagues^46^, we obtained Olink Normalized Protein eXpression (NPX) data and associated patient metadata from www.olink.com/mgh-covid-study. Of the 383 individuals included in this study, we removed patients without documented COVID-19 positivity, leaving 305 patients. In assessing the relationships of ADAMTS13 and FSTL3 with days post diagnosis, WHO severity, and 28-day survival we applied linear regression models and provide the overall model fit. For the regressions on WHO severity and 28-day survival we used only protein abundances in NPX units from D0 patient samples.

Between-group comparisons in patient demographics and outcomes were examined using analysis of variance (ANOVA), followed by pairwise comparisons using chi-squared, t-tests, or Fisher’s exact tests as appropriate between those with and without COVID-19, as well as those with and without cardiac involvement within each disease severity stratum. Pearson correlations were used to measure correlation of candidate proteins with cardiac biomarkers.

Analysis of the golden Syrian hamster lung RNA sequence data was done using STAR version 2.7.3a with the MesAur1.0 (GCF_000349665.1) assembly and annotation of the hamster downloaded from NCBI. Transcript abundance estimates were calculated internal to the STAR aligner using the algorithm of htseq-count. DESeq2 was used for normalization, producing both a raw and normalized read count table. Differential expression at the gene level were performed by DESeq2 implemented in the DESeq2 R package. An adjusted p-value < 0.05 was used to determine genes that were significantly upregulated or downregulated by SARS-CoV2 at day 4 post challenge compared to naïve controls using the Benajmini & Hochberg method.

## Supplementary Material

Supplement 1

Supplement 2

## Figures and Tables

**Table 1:** Clinical Characteristics of Subjects in Discovery Study

COVID-	Moderate COVID-19		Severe COVID-19			
Control (N=26)	CV− (N=13)	CV+ (N=12)	CV_low_ (N=14)	CV_high_ (N=15)	P_ANOVA_	
Age, years	62 (10)	63 (8)	72 (16)	62 (12)	63 (10)	0.11
Women	10 (39%)	9 (69%)	6 (50%)	4 (29%)	4 (27%)	0.18
Race	*					0.29
White	19 (73%)	6 (46%)	8 (67%)	5 (36%)	7 (47%)	
Black	1 (4%)	2 (15%)	2 (17%)	4 (29%)	5 (33%)	
Hispanic	2 (8%)	4 (31%)	2 (17%)	4 (29%)	3 (20%)	
Other	4 (15%)	1 (8%)	0 (0%)	1 (7%)	0 (0%)	
BMI, kg/m^2^	33.1 (4.1)*	27.6 (4.1)	31.1 (8.7)	27.9 (4.3)	31.9 (10.3)	0.06
**Past Medical History**						
Pulmonary disease	8 (31%)	2 (15%)	3 (25%)	4 (29%)	3 (20%)	0.85
COPD	0(0%)	1(8%)	0 (0%)	1(7%)	2 (14%)	0.34
Asthma	1 (4%)	2 (15%)	2 (17%)	2 (14%)	1 (7%)	0.63
ILD	0 (0%)	0 (0%)	1 (8%)	0(0%)	0 (0%)	0.22
CAD	0(0%)*	1 (8%)	5 (42%)	1 (7%)	3 (20%)	0.006
MI	0 (0%)	0 (0%)	3(25%)	0 (0%)	2(13%)	0.02
Stroke	0 (0%)	1 (8%)	1 (8%)	0 (0%)	2 (13%)	0.31
Hypertension	13 (50%)	6 (46%)	10 (83%)	10 (71%)	9 (60%)	0.23
Hyperlipidemia	11 (42%)	8 (62%)	7 (58%)	7 (50%)	9 (60%)	0.73
Diabetes Mellitus	1 (4%)*	2(15%)	6 (50%)	6 (43%)	7 (47%)	0.003
Cancer	0 (0%)*	3 (23%)	3 (25%)	0 (0%)	6 (40%)*	0.003
Liver Disease	2 (8%)	4 (31%)	1 (8%)	2 (14%)	2 (13%)	0.37
Kidney Disease	0 (0%)*	1 (8%)	6 (50%) *	1 (7%)	1 (7%)	<0.0001
**Baseline Medications**						
Anticoagulation	1 (4%)	0 (0%)	5 (42%)*	0 (0%)	3 (20%)	0.002
NSAID	9 (35%)	3 (23%)	5 (38%)	3 (21%)	8 (53%)	0.36
Statin	11 (42%)	5 (38%)	7 (58%)	6 (43%)	6 (40%)	0.86
Immunosuppression	0 (0%)	0 (0%)	1(8%)	1 (7%)	1 (7%)	0.56

Values are mean (SD) or n (%). P_ANOVA_ tested differences between groups.

* indicates P<0.05 on Fisher exact pairwise testing between COVID-19 patients and COVID-19 negative controls – groupings or between CV (+/−) or CV (low/high) within moderate and severe COVID-19 groups, respectively. CV = cardiac involvement.

## Data Availability

All data that support the findings of this study are included in the published article and its supplementary information files. The datasets during the current study are available from the corresponding author on reasonable request.
