# Supplementary material for: Plasma Proteomics of COVID-19 Associated Cardiovascular Complications: Implications for Pathophysiology and Therapeutics
Source: Res Sq. 2021 Jun 8:rs.3.rs-539712. Preprint. [Version 1] doi: 10.21203/rs.3.rs-539712/v1 (PMC8202429; doi:10.21203/rs.3.rs-539712/v1)
Supplement: Supplement 2 [file a64f512f1f2addf18b8aa3f7.docx]

**SUPPLEMENTARY INFORMATION**

**Plasma Proteomics of COVID-19 Associated Cardiovascular Complications: Implications for Pathophysiology and Therapeutics**

Jason D. Roh, MD, MHS^1^, Robert R. Kitchen, PhD^1^, J. Sawalla Guseh, MD^1^, Jenna N. McNeill, MD^2^, Malika Boudries, PhD^3^, Amanda Martinot, DVM, MPH, PhD^3,4^, Andy Yu, BSc^1^, Colin Platt, PhD^1^, James Rhee, MD, PhD^1,5^, Brittany Weber, MD, PhD^6^, Lena E. Trager, BA^1^, Margaret H. Hastings, PhD^1^, Sarah Ducat, BS^4^, Peng Xia, PhD^1^, Claire Castro, PhD^1^, Bjarni Atlason^1^, Timothy W. Churchill, MD^1^, Marcelo F. Di Carli, MD^6,7^, Patrick T. Ellinor, MD, PhD^1,8^, Dan H. Barouch, MD, PhD^3,9^, Jennifer E. Ho, MD^1^, Anthony Rosenzweig, MD^1^

Affiliations

^1^ Corrigan Minehan Heart Center, Division of Cardiology, Cardiovascular Research Center, Department of Medicine, Massachusetts General Hospital, Harvard Medical School, Boston, MA.

^2^ Division of Pulmonary and Critical Care, Department of Medicine, Massachusetts General Hospital, Boston, MA.

^3^ Center for Virology and Vaccine Research, Beth Israel Deaconess Medical Center, Boston, MA USA.

^4^ Department of Biomedical Sciences, Section of Pathology, Tufts University Cummings School of Veterinary Medicine, North Grafton, MA, USA.

^5^ Department of Anesthesia, Critical Care, and Pain Medicine, Massachusetts General Hospital, Boston, MA, USA.

^6^ Division of Cardiovascular Medicine, Department of Medicine, Brigham and Women’s Hospital, Harvard Medical School, Boston, MA.

^7^ Department of Radiology, Brigham and Women’s Hospital, Boston, MA, USA.

^8^ Cardiovascular Disease Initiative, The Broad Institute of MIT and Harvard, Cambridge, MA

^9^ Ragon Institute of MGH, MIT, and Harvard, Cambridge, MA, USA

**Supplementary Table 1: Laboratory values and outcomes in COVID-19 patients.** Median (IQR) or n (%). P_ANOVA_ tested differences between groups. * indicates P<0.05 on Fisher exact pairwise testing between CV(+/-) or CV (low/high) within moderate and severe COVID-19 groups, respectively. CV = cardiac involvement.

|  | **Moderate COVID-19** | | **Severe COVID-19** | |  |
| --- | --- | --- | --- | --- | --- |
|  | **CV- (N=13)** | **CV+ (N=12)** | **CV_low_ (N=14)** | **CV_high_ (N=15)** | **P_Anova_** |
| **Laboratory Values** |  |  |  |  |  |
| Trop T (ng/L) N=53 | 6 (5, 7) | 34 (24, 46)* | 10.5 (8, 14) | 69 (40, 160) | 0.06 |
| NTproBNP (pg/mL)  N=42 | 49 (36, 152) | 2084 (494, 5273)* | 86 (51, 296.5) | 3640 (2714, 6624)* | 0.005 |
| CRP (mg/L) N=53 | 27 (12, 66) | 97 (32, 159) | 75 (32, 124) | 172 (89, 256)* | 0.0001 |
| Initial Ddimer (ng/mL) N=52 | 784.5 (550, 1663.5) | 1195 (714, 1847) | 1603 (773, 2219) | 4000 (4000, 8848)* | <0.0001 |
| Peak Ddimer (ng/mL) N=53 | 1321 (351, 4000) | 2369 (848, 3408) | 3821(544, 10000) | 4833 (3750, 10000) | 0.0001 |
| Peak IL6 (pg/mL) N=28 | 35.2 (16.4, 67.9) | 66.3 (11.2, 94.3) | 63.2 (35.9, 120) | 58.2 (40.1,199) | 0.56 |
| Initial Ferritin (ug/L) N=52 | 652 (366, 1219) | 436 (203, 961) | 790(425, 1262) | 962 (558, 2267) | 0.53 |
| Peak Ferritin (ug/L) N=52 | 828 (423, 1888) | 907 (248, 1137) | 1313 (780, 1861) | 1206 (856, 4545) | 0.43 |
| **Outcomes** |  |  |  |  |  |
| Intubation | 0 (0%) | 0 (0 %) | 9 (64%) | 13 (87%) | <0.0001 |
| Vasopressor Use | 0 (0%) | 0 (0%) | 7 (50%) | 15 (100%)* | <0.0001 |
| Neuromuscular  blockade | 0 (0%) | 0 (0%) | 2 (14%) | 7 (47%) | 0.001 |
| Sedation | 0 (0%) | 0 (0%) | 9 (64 %) | 13 (87%) | <0.0001 |
| Renal replacement  therapy (RRT) | 0 (0%) | 0 (0%) | 1 (7%) | 8 (53%)* | <0.0001 |
| Anticoagulation | 2 (15%) | 4 (33%) | 2 (14%) | 11 (73%)* | 0.001 |
| PE | 1 (8%) | 0 (0%) | 1 (7%) | 1 (7%) | 0.83 |
| DVT | 2 (15%) | 0 (0%) | 0 (0%) | 2 (13%) | 0.24 |
| Death | 0 (0%) | 1 (8%) | 1 (7.1%) | 5 (33%) | 0.05 |

**Supplementary Figure 1: Mendelian randomization study design.** The flow diagram depicts the MR procedure used in our study. Phenoscanner was used to acquire summary levels SNPs associated with ADAMTS13 plasma protein levels (pQTLs). 129 SNPs were identified with locus wide significance (P < 0.05/129 = 3.88 x 10^-4^). Effect prioritized SNPs were thinned for independence leaving 6 SNPs for a multivariant genetic instrument. The 6 SNPs, their effect size (beta), standard error (se), effect and other alleles are tabulated. This genetic instrument for ADAMTS13 was used as the exposure. The UKB-b: 11309 dataset was used to interrogate outcomes with the diagnosis code I24.8 utilized as a proxy for myocardial injury.


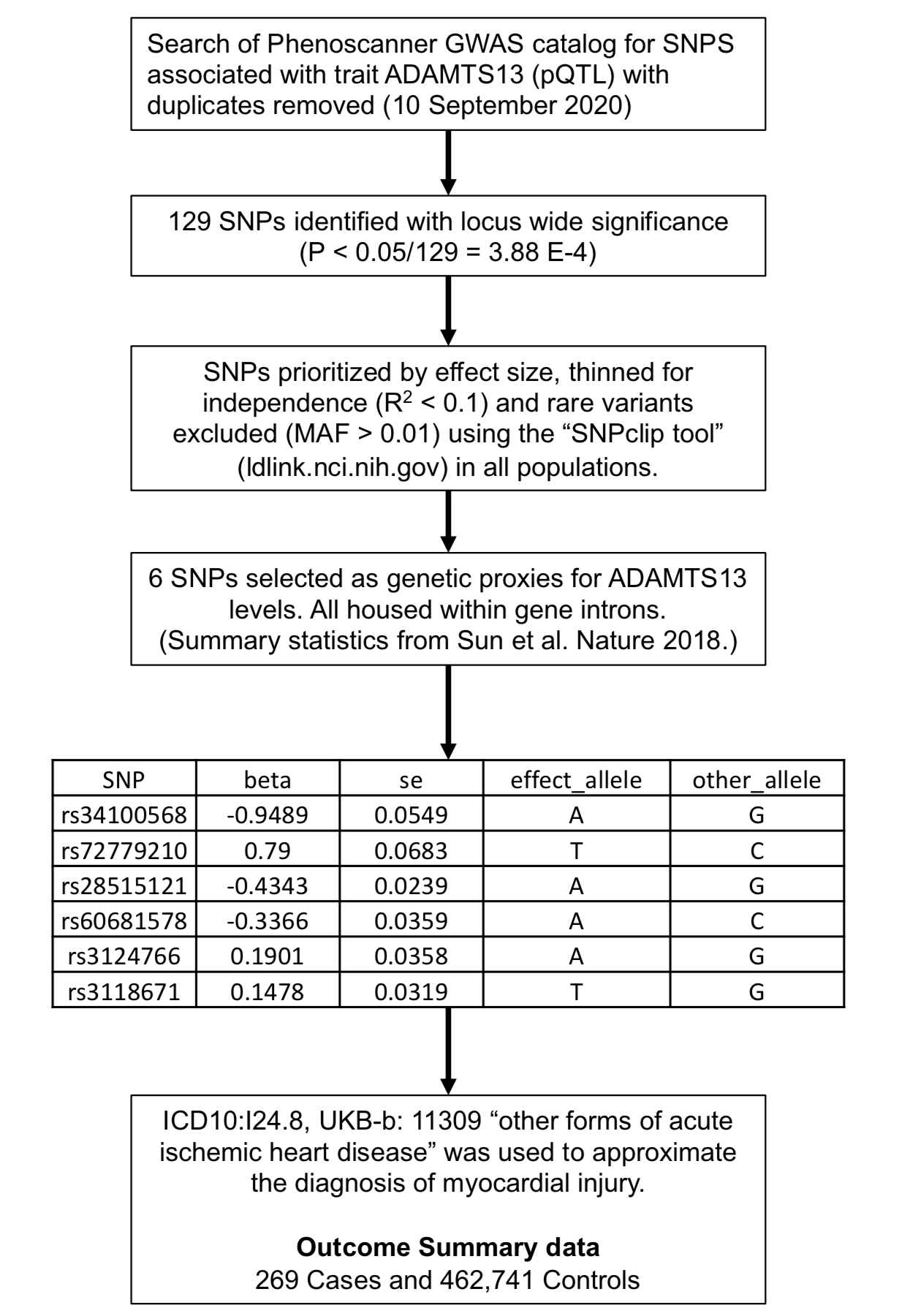


**Supplementary Figure 2: Top 25 upregulated and downregulated PC1 plasma proteins in the discovery cohort.** In the discovery study dataset, principal component 1 (PC1) distinguishes subjects according to COVID-19 disease severity and cardiac involvement (Fig. 1a). Data for the PC1 proteins with the greatest differential expression are shown as Tukey’s boxplots with median, inter-quartile range, and whiskers representing at most 1.5x inter-quartile range. RFU = relative fluorescence unit.

**Supplementary Figure 3: vWF/ADAMTS13 ratios according to prespecified subgroups in the discovery cohort.** Data are shown as boxplots with median, inter-quartile range, and minimum and maximum data points displayed. One-way ANOVA with post-hoc Tukey’s test was done with p < 0.05 considered significant. * p<0.05; ** p<0.01; ***p<0.001. CV= cardiac involvement

**Supplementary Figure 4: Correlation of plasma ADAMTS13 and d-dimer levels in the discovery cohort.** In the discovery study, 40 of the 56 COVID-19 patients had d-dimer levels, a biomarker of thrombotic burden, below the upper limit of the clinical assay (< 10,000 ng/ml). Pearson correlation was performed between ADAMTS13 somamer levels and clinical D-dimer levels. Solid line represents the best fit line from simple linear regression with the dashed line representing the 95% confidence interval. A p value < 0.05 was considered significant.

**Supplementary Figure 5: Correlation of SOMAmer cardiac TnT with clinical hsTnT levels in COVID-19 patients**. In the discovery study, subject’s SOMAmer cardiac TnT level (SOMAmer ID: SL000052) was correlated to the clinical high-sensitivity cardiac TnT level measured near the time of plasma sample collection (n=54 COVID-19 patients). Pearson correlation was performed with p < 0.05 considered significant.

**Supplementary Figure 6: Correlation of plasma ADAMTS13 levels with cardiac TNNI3 levels in the validation study.** In the validation cohort of 305 COVID-19 patients, Pearson correlation was performed on plasma ADAMTS13 NPX levels at day 0 (day of presentation to the MGH Emergency Department) with cardiac troponin I (TNNI3) NPX levels. P=2.1x10^-14^ considered significant. NPX = normalized protein expression.


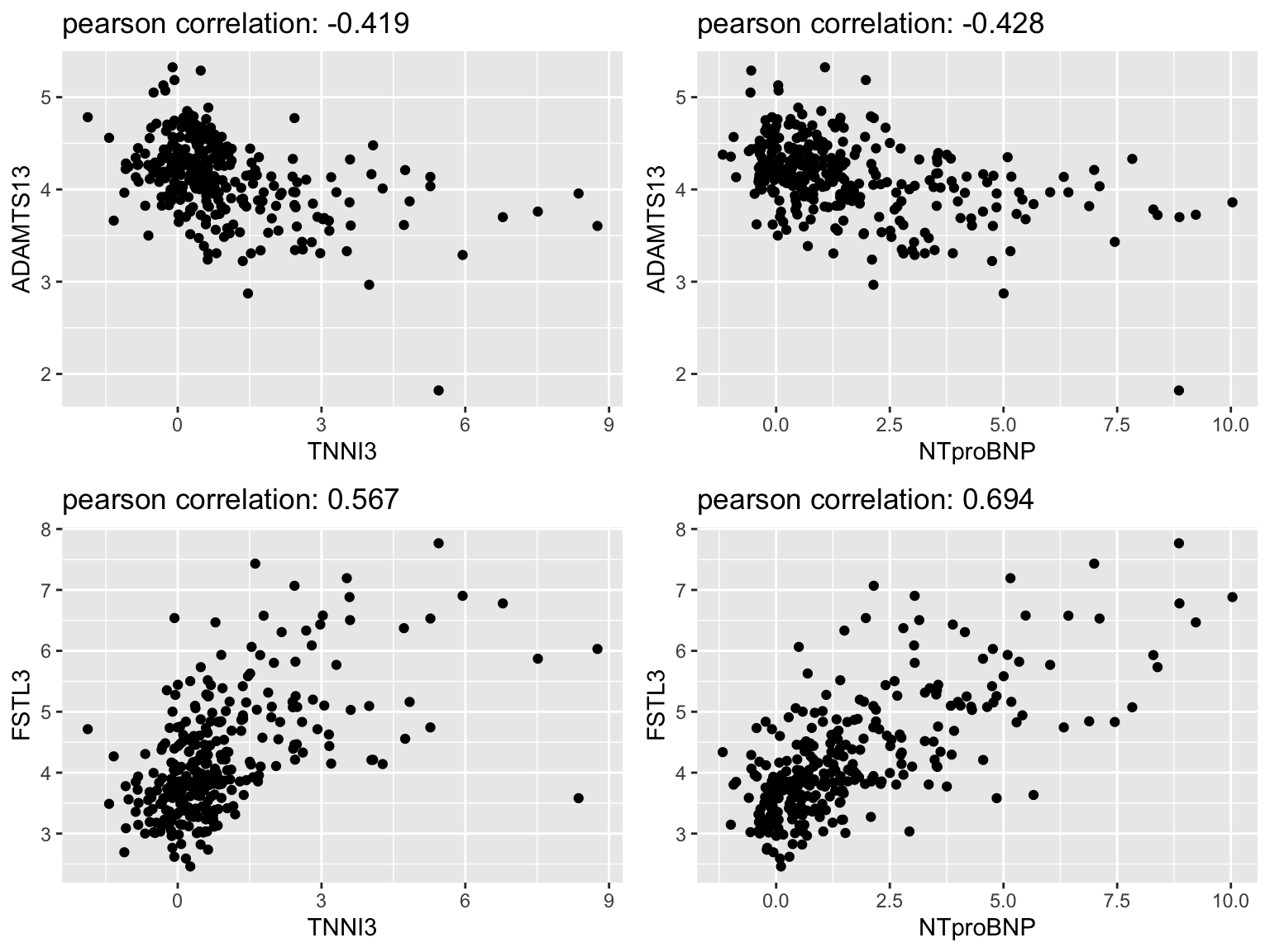


**Supplementary Figure 7: ADAMTS13 sensitivity analysis for myocardial injury.** **a)** The IVW estimate as well as the weighted median and MR Egger estimates are shown. The weighted median estimate only requires > 50% of SNPs to be valid. The MR Egger estimate demonstrated a consistant estimate but was not significant. A non-significant Egger Regression intercept (p = 0.937) suggests horizontal pleiotropy is not present and supports the validity of the IVW estimate. **b)** Single SNP analysis reveals the Wald ratio effect estimates using each single SNP. The MR estimates for the Egger and IVW methods are shown in red. **c)** A funnel plot to assess heterogeneity is shown. **d)** Leave-one-out sensitivity analysis is shown to indicate whether any given SNP has disproportionately influenced the result. Each black point on the forest plot represents the IVW method estimate with the given SNP excluded. The overall analysis is shown in red at the bottom for comparison.

**
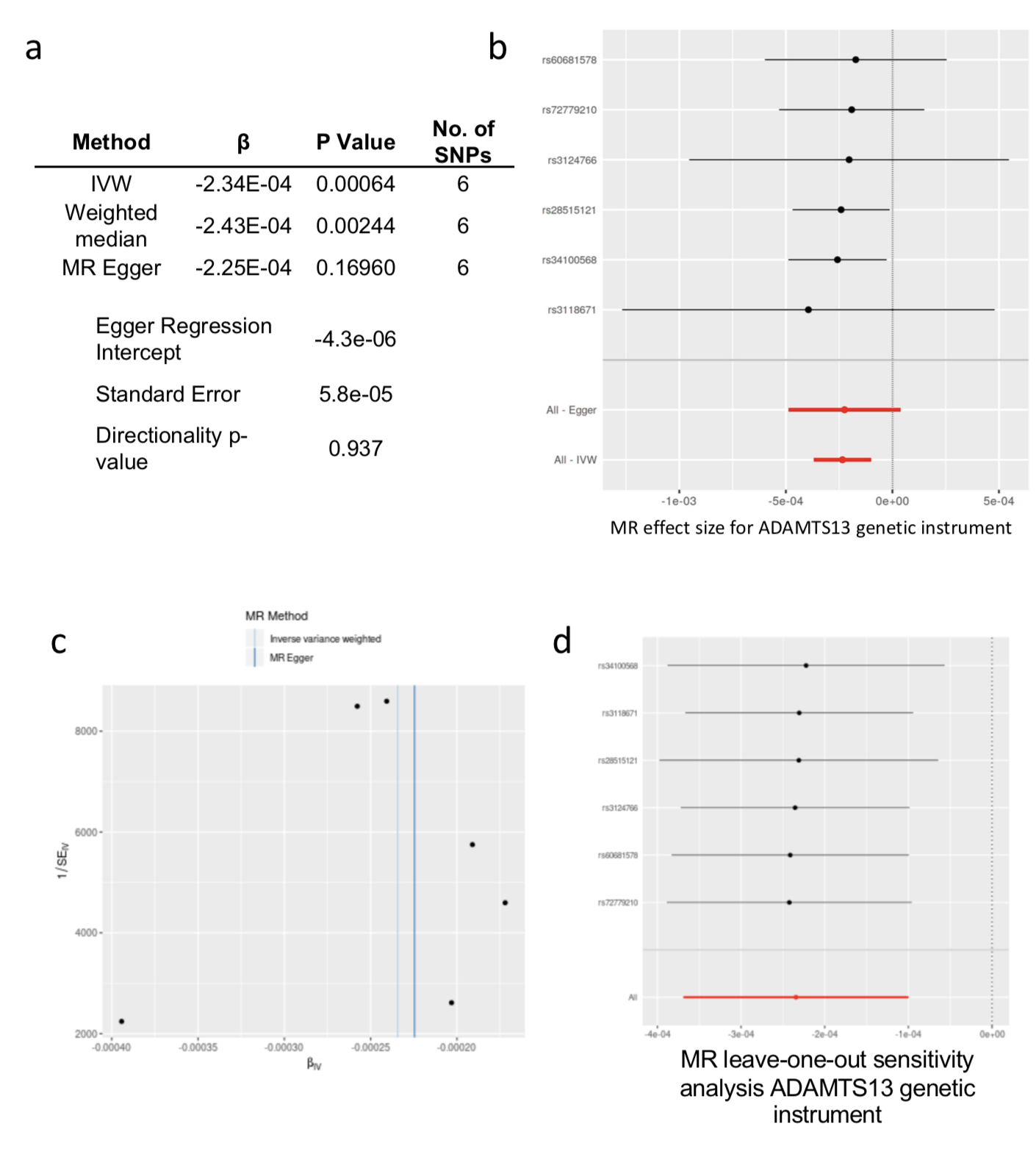
**

**Supplementary Figure 8: Correlation of NTproBNP SOMAmer with clinical NTproBNP levels in COVID-19 patients**. Subject’s SOMAmer NTproBNP level (SOMAmer ID: SL002785) was correlated to the clinical NTproBNP level measured near the time of the plasma sample collection. Forty-three of the 54 COVID-19 patients had clinical NTproBNP levels available. Pearson correlation was performed with p < 0.05 considered significant.

**Supplementary Figure 9: Correlation of plasma FSTL3 levels with cardiac TNNI3 and NTproBNP levels in the validation study.** In the validation cohort of 305 COVID-19 patients, Pearson correlation was performed on plasma FSTL3 NPX levels at day 0 (day of presentation to the MGH Emergency Department) with cardiac TNNI3 NPX levels or NTproBNP NPX levels. P<2x10^-16^ for both correlations. NPX = normalized protein expression.


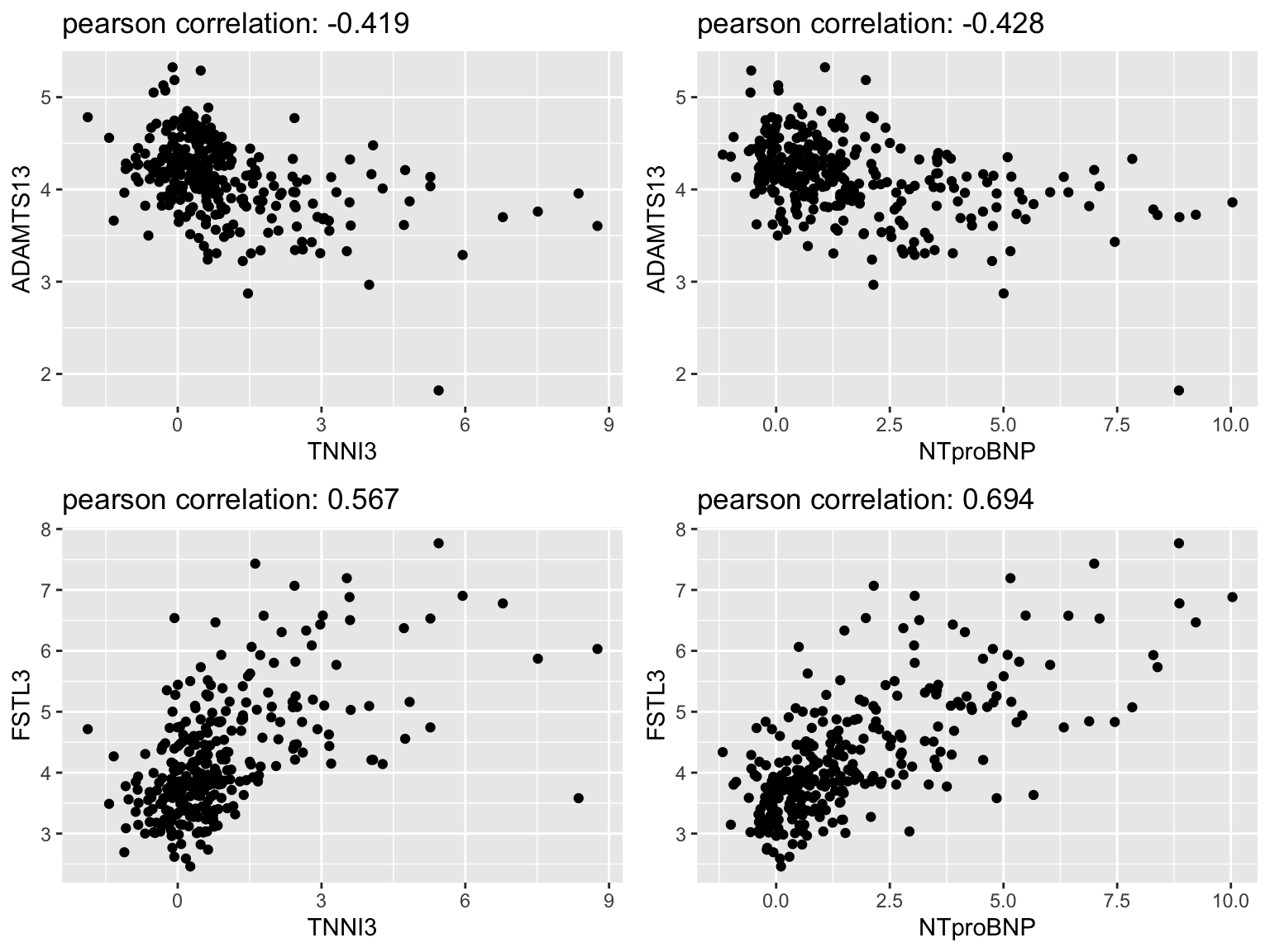


**Supplementary Figure 10: Correlation of TGFβ receptor and Activin receptor ligands with FSTL3.** Available SOMAmer levels of ligands capable of binding Activin or TGFβ receptors were correlated to FSTL3 SOMAmer levels (Koncarevic et al, Endocrinology, 2012). Pearson correlation was performed with p < 0.05 considered significant.

**Supplementary Figure 11: Induction of cardiomyocyte FSTL3 mRNA expression by various Activin and TGFβ receptor ligands.** Ligands that positively correlated with FSTL3 expression in the plasma proteomics dataset were individually tested to see if they increased FSTL3 expression in cardiomyocytes. Neonatal rat ventricular cardiomyocytes were incubated with recombinant human ligands (10 ng/ml x 18 hours). All proteins were obtained from either R&D Systems or Peprotech. RNA was extracted from cells using Trizol, and polymerase chain reaction was done using SYBR green and standard amplification protocols. Relative changes in FSTL3 mRNA expression were measured using the ΔΔCT method. The experiment was repeated four independent times (n=3-4/group/experiment). Data is displayed as mean ± standard deviation with each datapoint representing the mean from an experimental run. The black bar and closed points represent data from the controls while grey bars and open points represent data from the experimental treatment groups. For statistical analysis, the average fold change from each independent experiment was used as a single datapoint, and one-way ANOVA with Dunnett’s multiple comparison testing performed. *p<0.05; **p<0.01; ***p<0.001

***

***

*

**Supplementary Figure 12: Upregulation of TGFβ1 signaling in Syrian hamster model of COVID-19.** Heat map showing the significantly upregulated genes in the “Karlsson_TGFB1_Targets_Up” gene set. Only significant genes (p_adj_ < 0.05 after Benjamini Hochberg correction) are displayed in the heat map.

**
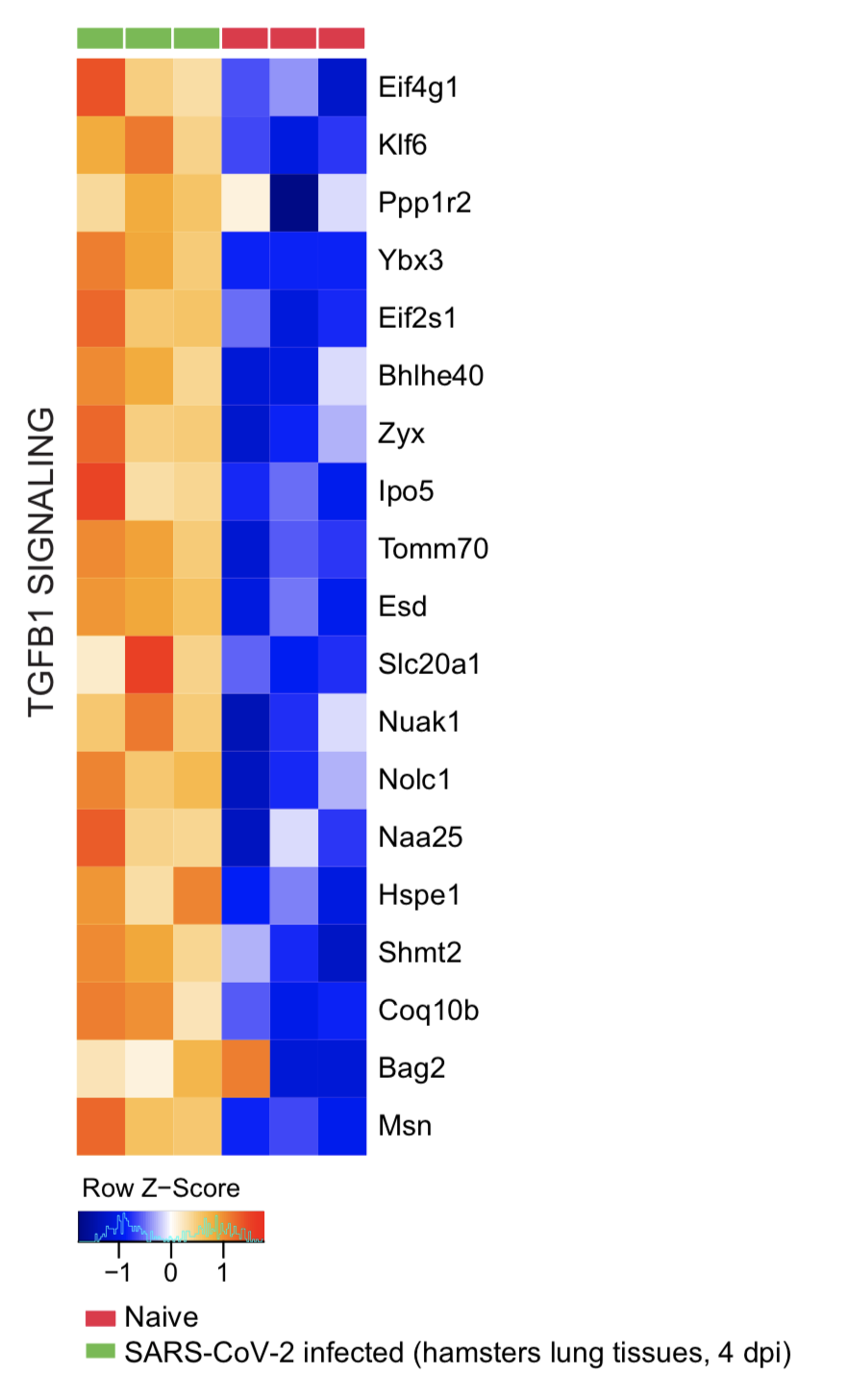
**
